# Serological Detection of Antibodies to Peste des Petits Ruminants Virus in Large Ruminants

**DOI:** 10.1111/tbed.12392

**Published:** 2015-07-22

**Authors:** M. Abubakar, M. Mahapatra, M. Muniraju, M. J. Arshed, E. H. Khan, A. C. Banyard, Q. Ali, S. Parida

**Affiliations:** ^1^ National Veterinary LaboratoryIslamabadPakistan; ^2^ The Pirbright InstituteWokingSurreyUK; ^3^ FAO FMD Project (GCP/PAK/123/USA)IslamabadPakistan; ^4^ Animal and Plant Health AgencyWeybridgeSurreyUK

**Keywords:** peste des petits ruminants virus, morbillivirus, serosurveillance, disease eradication, cattle, buffaloes

## Abstract

Peste des petits ruminants (PPR) is an economically important disease of small ruminants with a rapidly expanding geographical distribution. Peste des petits ruminants virus may manifest in a variety of ways with disease ranging from acute to subclinical. We investigated the exposure of large ruminants to PPRV in areas where the virus is endemic in the small ruminant population by assessing the serological status of groups of animals. This study focused on the Punjab province of Pakistan as an area where the virus is endemic and where mixed farming practices occur enabling close interactions between small and large ruminant populations. An overall PPR seropositivity was detected in 10.0% of cattle and 14.16% of buffaloes. Following an assessment of serological profiles in large ruminants within different age groups, a maximum seroprevalence was observed in cattle (17.5%) and buffaloes (22.5%) over 2 years of age indicating the potential utility of sampling large ruminant populations for PPR serosurveillance. The large ruminants sampled between one and two years of age had similar levels of seropositivity within populations with 11.2% and 16.2% of animals being seropositive, respectively. Current PPR vaccination strategies do not enable the differentiation between infected and vaccinated small ruminants, and as such, the serological surveillance of sheep and goats is of little value. When considering eradication programmes for PPRV, this factor is of great significance. However, where large and small ruminants are farmed together, serological surveillance of large ruminants may provide a snapshot of virus infection within populations where mild disease is present or where small ruminants are regularly vaccinated.

## Introduction

Peste des petits ruminants (PPR) is a highly contagious and acute viral disease of sheep and goats that causes great economic losses due to the high levels of morbidity and mortality often observed. The causative agent, PPR virus (PPRV), belongs to the genus *Morbillivirus* in the family *Paramyxoviridae* alongside *measles virus* (MV), *rinderpest virus* (RPV), *canine distemper virus* (CDV), *phocine distemper virus* (PDV), *porpoise morbillivirus* (PMV), *dolphin morbillivirus* (DMV) and recently identified novel morbilliviruses infecting cats (FeMV), bats and rodents (Drexler et al., 2012; Woo et al., 2012). Peste des petits ruminants virus typically manifests in small ruminants causing pyrexia, conjunctivitis, rhinotracheitis and ulcerative stomatitis, gastroenteritis and in severe cases, pneumonia (Taylor, 1984). Morbidity and mortality rates can be as high as 90–100% in naïve populations, dropping to nearer 20% in endemic areas (Banyard et al., 2010). Peste des petits ruminants virus is now endemic in most of Saharan and sub‐Saharan Africa, Turkey, the Middle East and the Indian subcontinent (Dhar et al., 2002; Banyard et al., 2010; Abubakar et al., 2011) and has recently been reported in areas previously thought to be free of the disease including Algeria, Morocco, China, the Democratic Republic of Congo (DRC), Sierra Leone and Tajikistan (De Nardi et al., 2012; WAHID Interface, 2011; Wang et al., 2009; Munir et al., 2012; Kwiatek et al., 2007; FAO, 2013; Banyard et al., 2014). Importantly, alongside causing devastating disease outbreaks, the virus is also able to circulate within small ruminant populations subclinically, often making detection very difficult. This feature of PPRV infection also makes serological assessment of circulating virus problematic, especially where small ruminant populations have been vaccinated as the serological differentiation between vaccinated and naturally infected animals is not currently possible (Sen et al., 2010). The highly contagious nature of PPRV and the distribution and movement of small ruminants creates a serious transboundary problem, inhibiting trade and heightening economic losses in affected areas, where small ruminants are often more important than cattle in food production (Banyard et al., 2010).

Following rinderpest eradication, measles and PPRV have also been identified as targets for potential global eradication. Indeed, capitalizing on the successful eradication of rinderpest, various international agencies including United Nations Food and Agriculture Organization (FAO) and the World Organization of Animal Health (OIE) are now focusing on PPR as the next veterinary virus for potential eradication. For any disease eradication programme, epidemiological surveillance is critical (Mariner et al., 2012). In addition, the role of species other than the established target species in the circulation and maintenance of the virus needs to be assessed to devise effective control strategies. Peste des petits ruminants virus serological positivity has historically been observed in large ruminant populations (Anderson and McKay, 1994; Abraham et al., 2005; Balamurugan et al., 2012, 2014; Lembo et al., 2013). Alongside this, reports detailing the isolation of live virus from subclinically infected cattle three weeks after transmission of the disease from experimentally infected goats have indicated the potential importance of domestic large ruminants in the dissemination of the virus and as indicators of circulating virus within populations (Lembo et al., 2013; Sen et al., 2014).

PPR is endemic across Pakistan where both organized farms and subsistence farming practices are established (Abubakar and Munir, 2014). Outbreaks of PPR in Pakistan between 1991 and 2013 are detailed in Table 1. In Pakistan, both large and small ruminants are generally farmed together within close contact, often sharing enclosed habitations as well as pasture and drinking areas. These farming practices provide ideal opportunities for the transmission of viruses between different small ruminant populations as well as between large and small ruminant populations. Here, we report the serological evidence of PPRV infection in cattle and buffaloes in the Punjab province of Pakistan and comment on the impact of the data on any future eradication campaign in small ruminants. Importantly, we highlight the utility of large ruminant serosurveillance as an indicator for the circulation of wild‐type virus in areas where small ruminants are regularly vaccinated.

**Table 1:** Reported outbreaks of Peste des petits ruminants virus (PPRV) within Pakistan

Year of PPR Outbreak	PPR outbreaks in different provinces of Pakistan	PPR outbreaks in Punjab province^a^	Type of evidence	Reference
1991	Punjab province	Faisalabad	Clinical and antigen detection	Athar et al. (1995)
1996	Punjab province	Not associated	Clinical and antigen detection	Amjad et al. (1996)
1997	Punjab province	Not associated	Clinical and antigen detection	Ayaz et al. (1997)
1998	Punjab province	Rawalpindi	Clinical and antigen detection	Hussain et al. (1998)
2002–2003	24 districts of all provinces	Attock, Mandi Bahaudin, Faisalabad	Clinical and antibody detection	Zahur et al. (2008)
2004	All provinces	Rawalpindi, Mandi Bahaudin, Attock, Bhakkar, Faisalabad	Antigen and antibody detection	Zahur et al. (2008); Abubakar et al. (2008)
2005	Various districts of Punjab, KPK and Sindh provinces (23 districts in Punjab)	All study districts except Jhelum & Narowal	Clinical and antibody detection	Khan et al. (2007); Zahur et al. (2011)
2006	All provinces (15 districts in Punjab)	Rawalpindi, Jhelum Pakpattan, Attock, Mandi Bahaudin, Faisalabad	Antigen and antibody detection	Khan et al. (2007); Abubakar et al. (2009); Durrani et al., 2010;
2007	6 districts of Punjab province	Rawalpindi	Clinical and antigen detection	Durrani et al. (2010)
2009	Sindh province	Not associated	Antibody detection	Abubakar et al. (2010)
2010	5 district of Punjab province	Faisalabad, Bhakkar, Attock	Antigen and antibody detection	Jalees et al. (2013)
2011	2 districts of Punjab province	Faisalabad	Antigen detection	Munir et al. (2012)
2013	3 districts of Punjab province	Rawalpindi, Attock	Antigen and antibody detection	Abubakar and Munir (2014)

a Indicates districts common between the studies; not associated indicates where there is no known association between outbreaks in districts presented in the current study and those previously documented.

## Materials and Methods

### Sampling plan

**Figure 1:**
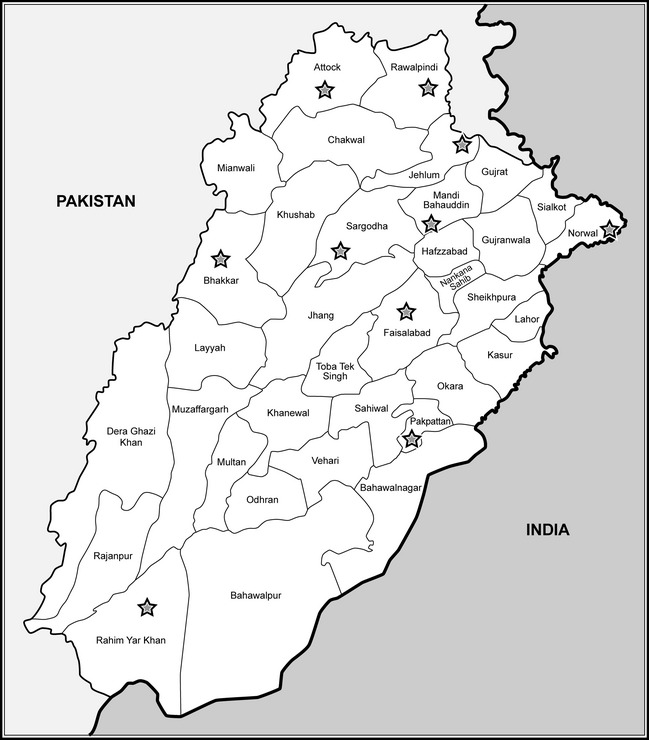
Districts of Punjab Province of Pakistan. The 10 districts where sampling took place are indicated by a star symbol.

Blood samples were collected from animals from 10 districts within the Punjab province of Pakistan between June and December 2009 (Table 2; Fig. 1). The ageing of animals was performed by examination of the incisor teeth in older animals and by recording the birth date of the young animals through interaction with farmers. Outbreaks of PPR in small ruminants across many districts of the Punjab province have been reported previously (Khan et al., 2007; Abubakar et al., 2008, 2009). However, no outbreaks were reported during 2008 and 2009 (Table 1), and the time of sample collection detailed in the present study. During this time, a two‐stage sampling design was followed in which the first stage was to select the villages to sample and the second stage was to select the animals to sample within each village. In each village, a maximum number of three animals were sampled from each household. Within the districts, households practiced mixed farming involving the housing and maintenance of large and small ruminants in close contact. Following this design, a total of 48 blood samples were collected randomly from each village taking an equal number from cattle and buffalo across each area (*n* = 24).

**Table 2:** District wise antibody seroprevalence in various age groups of cattle and buffaloes

Area	District	≤1 year (P/T)		1–2 year (P/T)		>2 years (P/T)	
		Cattle	Buffalo	Cattle	Buffalo	Cattle	Buffalo
North Punjab	Attock	0/8	0/8	2/8	2/8	1/8	0/8
	Jhelum	0/8	0/8	1/8	2/8	1/8	2/8
	Rawalpindi	0/8	0/8	1/8	0/8	2/8	2/8
Central Punjab	Bhakkar	1/8	0/8	0/8	1/8	2/8	1/8
	Faisalabad	0/8	0/8	0/8	3/8	3/8	5/8
	Mandi Bahaudin	0/8	0/8	0/8	0/8	0/8	1/8
	Narowal	0/8	0/8	1/8	3/8	1/8	2/8
	Sargodha	0/8	0/8	3/8	0/8	2/8	0/8
South Punjab	Pakpattan	0/8	1/8	1/8	2/8	1/8	3/8
	Rahim Yar Khan	0/8	2/8	0/8	0/8	1/8	2/8
Total positive		1/80	3/80	9/80	13/80	14/80	18/80

P – total no. positive, T – total no. tested.

### Grouping of animals

Following sampling, the serum samples were divided according to the age of the animals into the following groups: (i) young animals less than one year of age, (ii) animals between one and two years of age and (iii) animals over two years of age.

### Sample testing

Each sample was tested for the presence of PPRV‐specific antibodies using the anti‐hemagglutinin (H) PPRV competitive ELISA (cH‐ELISA, BDSL, UK). The assays were performed and analysed following the manufacturer's instructions. Samples with percentage inhibition value >50% were considered positive as per Anderson and McKay (1994). All the tests were carried out in duplicate wells, and the borderline‐positive samples were repeated to confirm results. The average of two results was used in subsequent analysis. In addition, 200 bovine sera from Italy were tested using the cH‐ELISA to determine the specificity of the assay for cattle.

## Results and Discussion

The main aim of this study was to investigate the seroprevalence of PPRV in cattle and buffalo populations in an agro‐ecologically defined area of Punjab in Pakistan where PPR is endemic in small ruminant populations. The presence of PPRV‐specific antibody was demonstrated in both cattle and buffalo and is proposed as an indicator of virus circulation in areas where large and small ruminants are farmed together. This highlights a potential mechanism to screen large ruminant populations to determine the asymptomatic circulation of PPRV in small ruminant populations both in the presence and absence of local vaccination. This mechanism of screening large ruminants may aid efforts to detect circulating PPRV in the absence of a DIVA vaccine for PPRV, especially where coordinated vaccination campaigns have been initiated. Certainly, if regular vaccination is implemented in sheep and goats, serosurveillance of domestic large ruminants gives added value to routine PPR clinical surveillance not only in Pakistan, but also in many other PPR endemic countries where mixed farming practises are implemented. Alongside serological assessment, monitoring of large ruminant species in areas where PPRV is endemic in small ruminants may indicate any potential disease profiles that may manifest should PPRV evolve to cause clinical disease in these species. Transmission of PPRV from infected goats to cattle has been recently reported (Lembo et al., 2013; Sen et al., 2014), and PPRV antigen has been detected in lions (Balamurugan et al., 2012) and camels (Khalafalla et al., 2010). These reports suggest that PPRV can switch hosts (Khalafalla et al., 2010; Lembo et al., 2013; Muniraju et al., 2014; Sen et al., 2014) as postulated for other morbilliviruses following rinderpest eradication (De Swart et al., 2012). In this case, both small and large ruminants might be assessed serologically, to detect an eventual change in viral host–pathogen interaction towards those species.

**Figure 2:**
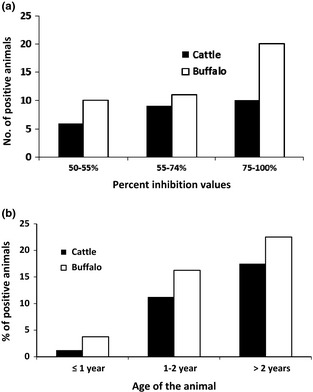
(a) Distribution of the Peste des petits ruminants virus (PPRV)‐specific antibodies in cattle and buffalo in Punjab province of Pakistan. (b) Age‐wise comparison of the PPRV antibodies in cattle and buffalo using cH‐ELISA.

The detection of serological evidence for PPRV infection of large ruminants across the districts was evaluated (Table 2). A large proportion of the samples were in the strong positive range (12.1%, 95% CI 9.4–15.3%) (Fig. 2a). Peste des petits ruminants seropositivity was detected in 10.0% (24/240) (95% CI 7.1–15%) of cattle and 14.16% (34/240) (95% CI 12.8–22.4%) of buffalo sampled in this study. These results indicated a higher PPRV seroprevalence in buffalo than in cattle within the populations studied; however, this was not statistically significant (*F*_1,479_ = 1.96, *P* = 0.162). Peste des petits ruminants outbreaks are generally seen to cause mild to moderate disease in sheep and goats in Pakistan due to the endemic situation. There have been occasional outbreaks of severe disease within Pakistan and although the drivers for differences in pathogenicity following infection with PPRV are ill‐defined, the introduction of virus to populations of naive animals often results in severe clinical disease.

As shown in Table 1, reports of PPR disease outbreaks in small ruminants were recorded in the years leading up to the sampling initiated in this study, suggesting the potential transmission of PPRV from small ruminants to large ruminants. Importantly, the detection of PPRV‐specific antibodies in cattle following laboratory confirmed outbreaks of PPRV in small ruminants has been reported previously (Lembo et al., 2013). Furthermore, Khan et al. (2008) reported PPRV serologic positivity in both cattle and buffalo (41.86% and 67.42%, respectively) in the Punjab province of Pakistan, although the basis for the high prevalence of PPR antibodies in large ruminants was unknown. In another study from the region (in the neighbouring country, India), Balamurugan et al. (2012) reported a 4.58% overall prevalence of PPRV antibodies in cattle and buffaloes across 2159 serum samples. A further study by Balamurugan et al. (2014) detected a slightly higher seroprevalence, 11.07% and 16.20% in cattle and buffaloes, respectively, across 1498 serum samples analysed.

In this study, to validate the serological positivity detected in large ruminant samples, 200 bovine sera from Italy were tested on the cH‐ELISA. All 200 samples were negative demonstrating a lack of false positivity seen with the assay when testing large ruminant sera. Whilst no test can be truly 100% specific, if the sample size is large enough, false positives may be detected. Indeed, the specificity of this test was at least as high as that described for the RPV‐based assay (99.9%). A similar level of specificity was reported by Couacy‐Hymann et al. (2007), using the same cH‐ELISA format with a sensitivity of 68% in sera derived from cattle. Historically, PPRV seropositivity in samples from cattle had been discounted due to the potential for circulating RPV antibodies that cross react with the PPRV ELISA. However, in the absence of RPV, the detection of large ruminants serologically positive for PPRV antibodies is of relevance to the epidemiology of PPRV. Of course, whilst rinderpest has been eradicated, the potential for the emergence of novel antigenically related ruminant morbilliviruses remains but this cannot be factored into a study of this nature.

Whilst the serological positivity of large ruminants for PPRV has been reported previously where outbreaks in small ruminants have occurred (Anderson and McKay, 1994; Abraham et al., 2005), this is the first assessment of serological positivity of large ruminants to PPRV based on the age of the animals sampled. Furthermore, in the present study, sampling was performed on healthy animals at a time when clinical disease in small ruminants had not been reported in the area. The presence of PPRV‐specific antibodies demonstrates that cattle and buffaloes are exposed to PPRV infection naturally following transmission from small ruminants although the possibility of horizontal transmission between large ruminants remains to be investigated.

The findings in the present study largely agree with previous reports regarding the seropositivity of large ruminant populations to PPRV. Certainly, the observation that in general, buffalo seroconvert more readily than cattle is reflected in several published manuscripts (Khan et al., 2008; Balamurugan et al., 2012, 2014). However, none of the previous reports assessed the effect of age on serological status in large ruminant populations. From the present study, it appears that calves aged <1 year were least likely to have seroconverted (1.2%, 1/80) (95% CI 0–7.4%) (Fig. 2b) although clarity regarding the passage of antibody protection derived from maternal antibodies is required. The greatest degree of seroconversion was observed in animals >2 years of age, in 17.5% (95% CI 10.6–27.4%) and 22.5% (95% CI 14.6–32.9%) of cattle (14/80) and buffalo (18/80) (Fig. 2b), respectively, perhaps reflecting the increased time period for potential exposure to the virus in the field. The animals that were sampled between one and two years of age had similar levels of seroconversion within populations with 11.2% of cattle (95% CI 5.8–20.2%) (9/80) and 16.25% of buffalo (95% CI 9.6–26%) (13/80) being seropositive, respectively. It is worth noting that there was no reported outbreak in the Punjab (Table 1) province within the 1–2 years preceding the sample collection for this study which could be the reason for the low prevalence of PPRV‐specific antibodies detected in <1‐ to <2‐year‐old cattle and buffaloes. In addition, statistical differences between the age groups of both cattle (*F*_2,958_ = 7.39, *P* = 0.001) and buffalo (*F*_2,958_ = 6.95, *P* = 0.001) were observed.

In conclusion, the assessment of serological profiles of large ruminants in areas where small ruminants have been vaccinated against PPR may serve as a useful tool for the detection of circulating PPRV in the absence of DIVA vaccines. Across many areas considered endemic for PPR, large and small ruminants are housed and reared together creating numerous opportunities for virus transmission.
